# Rapid and large-scale implementation of HCV treatment advances in France, 2007–2015

**DOI:** 10.1186/s12879-017-2889-4

**Published:** 2017-12-20

**Authors:** Cécile Brouard, Marjorie Boussac-Zarebska, Christine Silvain, Julien Durand, Victor de Lédinghen, Josiane Pillonel, Elisabeth Delarocque-Astagneau

**Affiliations:** 1 Santé publique France, the national public health agency, Saint-Maurice, France; 2 Hepatology Unit, University Hospital, Poitiers, France; 3 Investigation Centre of Liver Fibrosis, Haut-Lévêque Hospital, Bordeaux University Hospital, Pessac, France; 4 INSERM 1181, Biostatistics, Biomathematics, Pharmacoepidemiology, and Infectious Diseases (B2PHI), Paris, France; 5 Institut Pasteur, B2PHI, Paris, France; 6 Versailles Saint-Quentin University, UMR 1181, B2PHI, Montigny-le-Bretonneux, France

**Keywords:** Hepatitis C, Treatment, Direct-acting antivirals, Health insurance data, Epidemiology, France

## Abstract

**Background:**

The last decade was marked by major advances in HCV treatment with the introduction of first wave protease inhibitors (1st-wave PIs, telaprevir or boceprevir) in 2011 and second direct-acting antivirals (2nd-wave DAAs) in 2014, that followed low effective pegylated interferon α / ribavirin bitherapy. We estimated the number of patients initiating HCV treatment in France between 2007 and 2015 according to the type of therapy, described their demographical characteristics, and estimated how many were cured with 2nd-wave DAAs in 2014–2015.

**Methods:**

Individual data from the national health insurance information system were analysed. HCV treatment initiation was defined as a drug reimbursement in the absence of any reimbursement for the same drug in the previous six weeks.

**Results:**

Between 2007 and 2015, 72,277 patients initiated at least one HCV treatment. The annual number of patients initiating treatment decreased from 2007 (~13,300) to 2010 (~10,000). It then increased with the introduction of 1st-wave PIs (~12,500 in 2012), before decreasing again in 2013 (~8400). A marked increase followed upon the approval of 2nd-wave DAAs in 2014 (~11,600). Approximately, 8700 and 14,700 patients initiated 2nd-wave DAAs in 2014 and 2015, respectively, corresponding to an estimated 20,300 cured patients in 2014–2015.

Patients initiating HCV treatment were mostly male (~65% throughout the 9-year period). Women were older than men (mean age: 55.0 vs. 48.9). Increasing age was associated with more advanced treatment. Among patients initiating 2nd-wave DAAs, the proportions of those under 40 and over 79 years old increased between 2014 and 2015, whereas the proportion of those previously treated for HCV 2007 onwards declined.

**Conclusions:**

Successive advances in HCV treatment have been rapidly and widely implemented in France. With the announcement of universal access to DAAs in mid-2016 and price reductions, access to 2nd-wave DAAs is expected to expand even more.

## Background

With an estimated 71 million people chronically infected [1] and almost 700,000 annual deaths from liver cirrhosis and hepatocellular carcinoma (HCC) [2], Hepatitis C virus (HCV) infection constitutes a serious worldwide public health problem. However, recent studies have demonstrated a significantly lower risk of developing HCC, lower all-cause mortality, and better health-related quality of life in patients achieving sustained virological response (SVR) than in untreated patients and non-sustained virological responders [3, 4]. SVR, defined as undetectable HCV RNA 12 or 24 weeks after treatment completion, is the primary goal of HCV therapy [5].

Until 2011, HCV standard of care was the combination of pegylated interferon (PEG-IFN) α and ribavirin (RBV) for 24 or 48 weeks. It was associated with poor SVR (50–80% according to HCV genotype) and caused serious side effects, often resulting in treatment discontinuation. In 2011, the standard of care for genotype 1 became a triple therapy combining PEG-IFN, RBV and either of the first wave protease inhibitors (1st-wave PIs), namely telaprevir (TVR) or boceprevir (BOC). These two PIs constituted the first wave of direct-acting antivirals (DAAs). These new regimens achieved higher SVR than PEG-IFN/RBV bitherapy (~ 65–75%), but they had serious adverse effects and were very expensive [6, 7]. Since 2014, a second-wave of DAAs (2nd-wave DAAs), which are more effective and better tolerated, have been licensed. Initially they were used as part of triple combination regimens with PEG-IFN/RBV, then in IFN-free regimens with or without RBV [8, 9]. However, because of their high prices and the heterogeneity of incomes and health insurance systems across Europe, indications for prescribing 2nd-wave DAA-based regimens differ between countries [10, 11].

In France, DAAs were initially used primarily in clinical trials and in compassionate use programmes (CUP). CUP are early access programmes intended to facilitate the availability of new medicines to patients suffering from life threatening diseases who do not meet clinical trials inclusion criteria [12]. From 2014 - when European Marketing Authorisation (MA) was granted - until mid-2016 [13, 14], the use of 2nd-wave DAAs was restricted to HCV chronically-infected adult patients with: a) stage F3, F4 or “severe F2” liver fibrosis, or b) comorbidities (HIV coinfection, mixed cryoglobulinaemia, non-Hodgkin B cell lymphoma) irrespective of fibrosis stage [15]. For each patient, eligibility to initiate 2nd-wave DAA treatment was assessed by a multi-disciplinary team in HCV reference centres. Prescribed treatments were then dispensed in hospital pharmacies. Despite these restrictions, an estimated 20,000 to 30,000 annual patients were expected to be treated by 2nd-wave DAAs during the first years after license approval [16, 17].

France is a low endemic country for HCV, with an estimated viremic infection prevalence in the general population of 0.42% (95% Credibility Interval (CrI) 95%:[0.33–0.53]), corresponding to 192,700 persons (95% CrI:[150,935–246,055]) [18]. However, among this population, an estimated 74,102 individuals (plausible interval: 64,920–83,283) are undiagnosed [19], suggesting that the current French HCV screening strategy, which exclusively targets people at high risk of infection (intravenous or intranasal drug users, recipients of blood transfusion before 1992, people with HIV infection, prisoners, etc.), is only partially effective [16]. Anti-HCV antibody prevalence can reach 4.3% in migrants living in difficult socio-economic conditions [20], 4.8% (95% Confidence Interval (CI): 3.5–6.5) in prison inmates [21] and 64% [95% CI: 59.2–68.2] in drug users (DU) who have injected at least once in their lifetime [22]. New infections mainly occur in active injecting DU, where the estimated incidence is 11.2/100 person-years [23]. The most frequent genotypes (GT) are GT1 (56–61%), GT3 (19%) and GT4 (9–17%) depending on patient recruitment [24, 25].

In the context of recent major advances in HCV treatment, we aimed to: a) estimate the number of patients who initiated HCV treatment in France between 2007 and 2015 according to the type of therapy, b) describe the evolution of their demographical characteristics and c) estimate the number of patients cured with 2nd-wave DAA-based regimens in 2014 and 2015.

## Methods

### Data source

Individual data were extracted from the French national health insurance system (SNIIRAM) database, which has been gradually developed since 1999 [26, 27]. Created in 1945, France’s Social Security system comprises several schemes which cover various occupational sectors (salaried employees in the private sector, public employees, students, self-employed workers, agricultural workers, etc.). Dependents of employed people, retired persons, the unemployed, minimum social welfare beneficiaries and irregular migrants living in France for more than three months, with or without a fixed address, also have access to free social security care. Since 2009, SNIIRAM has collected individual data on all healthcare reimbursements for all people affiliated to health insurance schemes in France. Today it covers almost the entire population living in France (66 million in 2015) [27]. SNIIRAM also includes information from the programme for the medicalization of information systems (PMSI) of public and private hospitals, specifically data on hospital stays and on the expensive drugs dispensed, including drugs in CUP. Constitution of the SNIIRAM data warehouse is based on the reliable identification of individuals by the national identification number (NIR), under which each individual is registered for his/her lifetime. Data are available for age, gender, and whether or not the person is a beneficiary of Complementary Universal Health Insurance (CMUC, which is free insurance for low-income persons) or State Medical Assistance (AME, which is free insurance for low-income irregular migrants). Patients registered with a long-term disease (LTD) are 100% reimbursed for their health expenditures in France, and disease diagnosis is recorded in the SNIIRAM database. Patients with chronic HCV infection are eligible for LTD status (code B182 of the 10th International Classification of Disease) if they have a severe infection and/or need antiviral treatment or long-term follow-up [28].

### Definitions and data management

All the reimbursements for the following drugs, provided in private or hospital pharmacies between 2006 and 2015, were extracted with the dates of the drugs delivery, the names of the drugs and the pseudonymised NIR of the individuals: PEG-IFN α and RBV (simultaneous deliveries), TVR, BOC, or 2nd-wave DAAs, namely sofosbuvir (SOF), simeprevir (SMV), daclatasvir (DCV), sofosbuvir + ledipasvir (SOF + LDV), ombitasvir + ritonavir-boosted paritaprevir (OBV + PTVr), dasabuvir (DSV). The following variables of interest were also extracted from the SNIIRAM databases: age, gender, benefiting from CMUC, AME or LTD status for HCV.

**Fig. 1:**
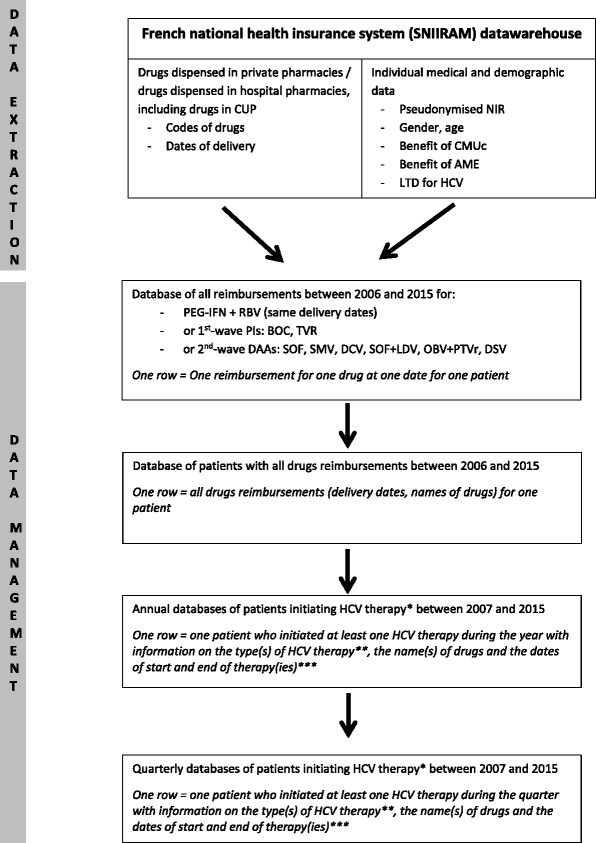
Schematic overview of the main steps of data extraction and management CUP: Compassionate use programme, NIR: National identification number, CMUC: Complementary Universal Health Insurance, AME: State Medical Assistance, LTD: Long-term disease, PEG-IFN: Pegylated Interferon α, RBV: Ribavirin, PIs: Protease inhibitors, BOC: boceprevir, TVR: telaprevir, DAAs: Direct-acting antivirals, SOF: sofosbuvir, SMV: simeprevir, DCV: daclatasvir, LDV: ledipasvir, OBV: ombitasvir, PTVr: ritonavir-boosted paritaprevir, DSV: dasabuvir. * Initiation of HCV therapy was defined as reimbursement for a drug in the absence of any reimbursement for the same drug in the six previous weeks. **The type of therapy was classified according the following algorithm: 1) “1st-wave PIs” in case of reimbursement of TVR or BOC + PEG-IFN α + RBV; 2) “2nd-wave DAAs” in case of reimbursement of SOF, SMV, DCV, SOF + LDV, OBV + PTVr, DSV +/− PEG-IFN α +/− RBV; 3) “PEG-IFN/RBV bitherapy” otherwise and in case of simultaneous deliveries of PEG-IFN α and RBV. *** The date of end of therapy was defined by the last delivery date without discontinuation (i.e. without a delay of more than six weeks between two reimbursements for the same drug). This date was not available for all patients initiating HCV therapy in 2015. Some patients may have initiated successive HCV treatments during the year or the quarter

Figure 1 describes the main steps of data management to obtain, from the database of drugs reimbursements, annual and quarterly databases of patients initiating HCV therapy between 2007 and 2015. Initiation of HCV therapy was defined as reimbursement for a drug in the absence of any reimbursement for the same drug in the previous six weeks. This delay corresponds to 1.5 times the usual interval between two HCV drug prescriptions (four weeks) in the absence of treatment discontinuation, and was determined after observing distributions of the delays between two deliveries for the same drug and following input from experts in hepatology. Using drugs’ names and dates of delivery, and following HCV treatment guidelines [5, 8, 9, 29], the type of therapy was classified according the following algorithm: 1) “*1st-wave PIs”* in case of reimbursement of TVR or BOC + PEG-IFN α + RBV; 2) *“2nd-wave DAAs”* in case of reimbursement of SOF, SMV, DCV, SOF + LDV, OBV + PTVr, or DSV, +/− PEG-IFN α +/− RBV; 3) *“PEG-IFN/RBV bitherapy”* otherwise and in case of simultaneous deliveries of PEG-IFN α and RBV.

### Statistical analysis

The number of patients initiating HCV therapy was calculated: a) by type of therapy (PEG-IFN/RBV bitherapy, 1st-wave PIs or 2nd-wave DAAs) for each year between 2007 and 2015; b) by drug for each quarter for patients initiating 1st-wave PIs or 2nd-wave DAAs between 2011 and 2015. As data for some insurance schemes were lacking for 2007 and 2008, the annual numbers of patients initiating PEG-IFN/RBV bitherapy during these years were extrapolated using data from 2009.

Demographical characteristics of patients initiating HCV treatment between 2007 and 2015 were described by type of therapy. Patients initiating 2nd-wave DAA-based regimens in 2015 were compared with those initiating 2nd-wave DAA-based regimens in 2014 and with those initiating PEG-IFN/RBV bitherapy in 2015 for the following variables: gender, age, benefitting from CMUC or AME, LTD status for HCV and previous HCV treatment initiation between 2007 and 2013.

The number of patients cured with 2nd-wave DAA-based regimens in 2014 and 2015 was estimated using the number of patients initiating these regimens during this two-year period and assuming an SVR rate of 90% in the real world [30, 31].

Statistical analyses were performed using SAS Enterprise Guide 4.3 software.

The SNIIRAM received approval from the French data protection authority (CNIL: Commission nationale de l’informatique et des libertés) in October 2001. A ministerial order dated 1st December 2011 gave to accredited staff of the national public health agency access to SNIIRAM anonymized individual data. The authors, who did the extraction and analysis, were accredited to Sniiram database access.

## Results

### Number of patients initiating HCV antiviral therapy

#### Annual evolution, 2007–2015

Between 2007 and 2015, 72,277 patients initiated at least one HCV treatment.

**Fig. 2:**
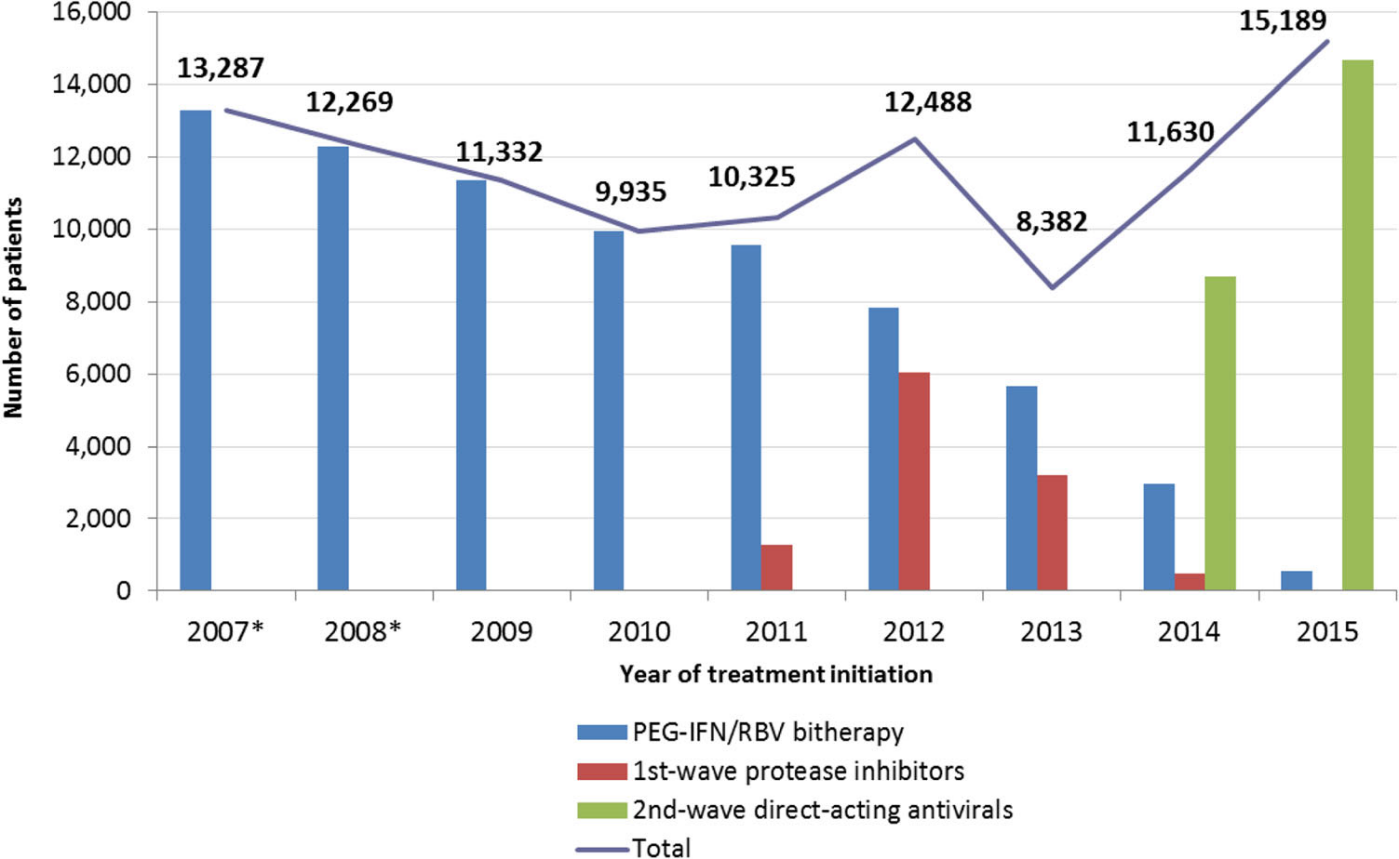
Annual number of patients initiating HCV antiviral therapy, France, 2007–2015 *Data were extrapolated for 2007 and 2008 to take into account the absence of data for some insurance schemes in the SNIIRAM database. The annual number of patients initiating a treatment (total) is less than the sum of the annual numbers of patients by type of therapy because some patients may have initiated successive HCV treatments with different types of therapies during the same year

The annual number of patients initiating HCV treatment progressively decreased between 2007 and 2010 from 13,287 to 9935 (Fig. 2). During this period, PEG-IFN/RBV bitherapy was the only available treatment. With the introduction of 1st-wave PI regimens, the annual number of patients initiating at least one treatment increased slightly in 2011 and more markedly in 2012, reaching 12,488. First-wave PI-based regimens concerned 1265 and 6037 patients in 2011 and 2012, respectively, corresponding to 12.3% and 48.3% of patients initiating treatment. A decrease was observed in 2013 (−33% compared with 2012) with only 8382 patients starting a treatment, 3199 (38.2%) of whom started a 1st-wave PI-based regimen. In 2014 and 2015, the annual number of patients initiating at least one HCV treatment increased sharply, reaching 11,630 and 15,189 patients, respectively. Second-wave DAA-based regimens concerned 8702 and 14,650 patients in 2014 and 2015, respectively, that is 74.8% and 96.5% of patients initiating treatment.

Over the 2007–2015 period, 53,794 patients initiated at least one treatment with PEG-IFN/RBV bitherapy, 10,260 with a 1st-wave PIs and 22,565 with a 2nd-wave DAAs. Assuming a SVR of 90%, 2nd-wave DAA-based regimens may have cured 20,309 patients.

#### Quarterly evolution, 2011–2015

**Fig. 3:**
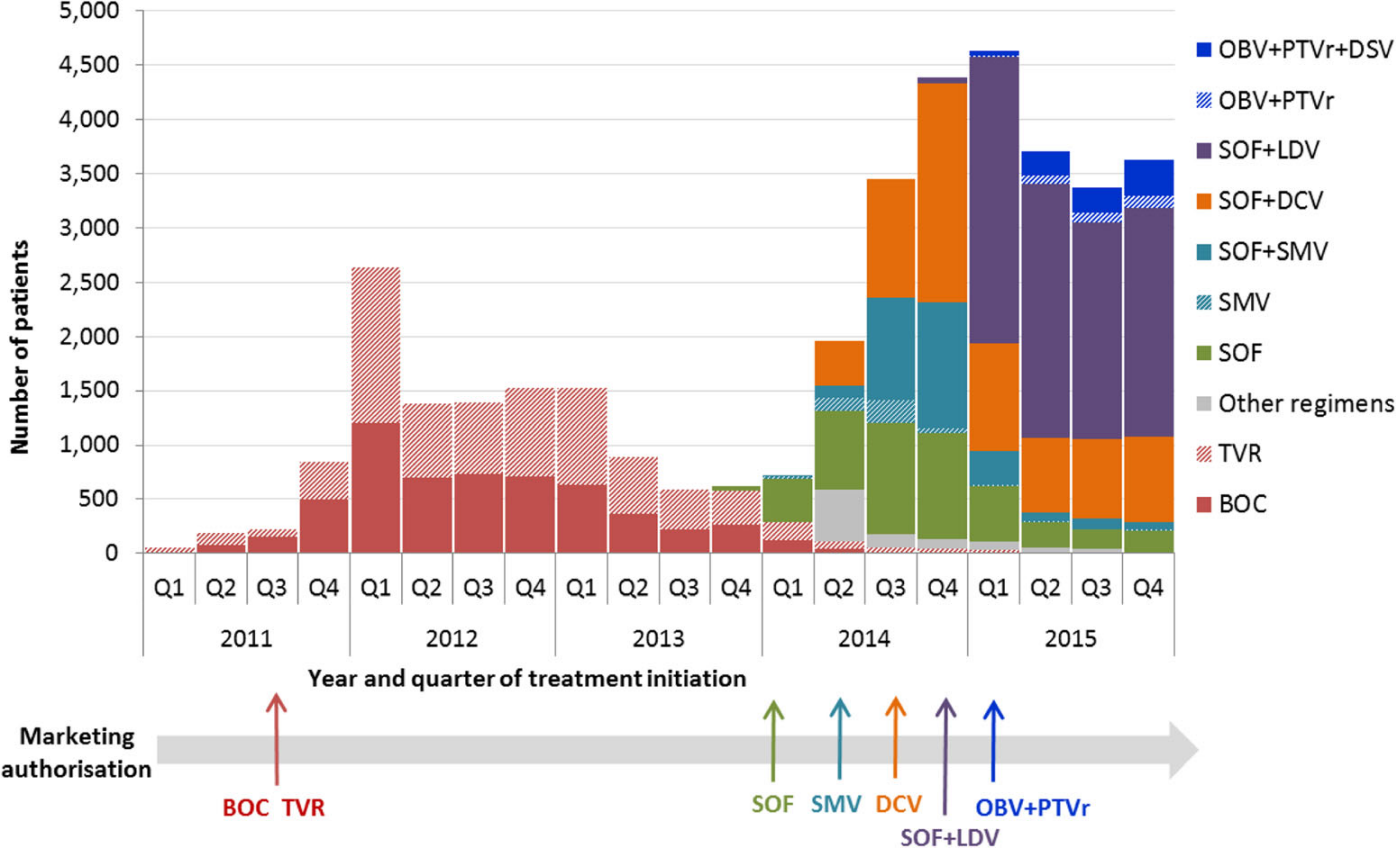
Quarterly numbers of patients initiating treatment with DAA-based regimens, France, 2011–2015 Q: quarter, BOC: boceprevir, TVR: telaprevir, SOF: sofosbuvir, SMV: simeprevir, DCV: daclatasvir, LDV: ledipasvir, OBV: ombitasvir, PTVr: ritonavir-boosted paritaprevir, DSV: dasabuvir

Quarterly numbers of patients initiating 1st-wave PIs or 2nd-wave DAA regimens are shown in Fig. 3.

The use of 1st-wave PIs was identified from the first quarter of 2011 (CUP in December 2010). After the MA for BOC and TVR obtained, respectively, in July and September 2011, an increase in the number of patients initiating regimens containing these drugs was observed in the last quarter of 2011, with a peak in the first quarter of 2012 (with 1204 and 1437 patients starting a regimen with BOC and TVR, respectively). Subsequently, the numbers of patients initiating 1st-wave PI-based regimens decreased, but remained between 600 and 900 for four quarters before a gradual decrease was seen until the beginning of 2015.

The use of 2nd-wave DAA-based regimens was identified for the first time in the last quarter of 2013 for SOF (CUP in September 2013) and the first quarter of 2014 for SMV alone or combined with SOF (CUP in October 2013). Patient numbers remained low (fewer than 50 per quarter) before the MA (in January 2014 for SOF and May 2014 for SMV), and then increased to 1024 patients for SOF in the 3rd quarter of 2014 and 1163 patients for SOF + SMV regimens in the final quarter. A limited number of patients used regimens containing SMV without SOF (max = 209 in the 3rd quarter of 2014). After CUP for DCV in March 2014, the number of patients initiating SOF + DCV regimens started to grow rapidly from the 2nd quarter (408 patients) to the MA in August 2014 and reached a peak (*n* = 2014) in the last quarter of 2014. In the 2nd quarter of 2014, a substantial number of patients (*n* > 400) were identified with DAA combinations other than those recommended (DCV+/-RBV for more than 97%). This number decreased sharply in the 3rd quarter with a gradual decrease during the following quarters. The end of 2014 was marked by concomitant CUP and MA for SOF + LDV in November which resulted in a large number of patients initiating SOF + LDV regimens (*n* = 2639) and a peak of patients initiating 2nd-wave DAA-based regimens in the first quarter of 2015 (*n* = 4637 patients). From the 2nd quarter of 2015, this total number decreased and remained steady at approximately 3500 per quarter until the end of 2015. The number of individuals initiating SOF + LDV regimens was stable at ~2000 per quarter whereas the number of those initiating other SOF-containing regimens tended to decline. People initiating OBV + PTVr ±DSV regimen were identified from the 1st quarter of 2015. Their numbers slightly increased throughout the year, reaching 451 patients in the last quarter.

In 2014, 89.8% and 66.3% of patients, respectively, initiated IFN-free and RBV-free 2nd-wave DAA-based regimens. In 2015, these proportions were 99.3% and 65.3%, respectively.

### Patients’ characteristics

#### Evolution by type of therapy, 2007–2015

The 72,277 patients initiating at least one treatment between 2007 and 2015 were predominantly male (65.6%) (Table 1). A rise in the proportion of men was observed among patients initiating PEG-IFN/RBV bitherapy, particularly from 2013 onwards. The proportions of men among patients initiating either 1st-wave PI-based regimens in 2012–2013 or 2nd-wave DAA-based regimens in 2014–2015 were lower than in patients initiating PEG-IFN/RBV bitherapy.

**Table 1:** Demographical characteristics of patients initiating HCV treatment by year of initiation and type of therapy, France, 2007–2015

Year	PEG-IFN/RBV bitherapy				1st- wave protease inhibitors				2nd- wave direct-acting antivirals				Total^b^			
	*n*	% men	Mean age (SD)		*n*	% men	Mean age (SD)		*n*	% men	Mean age (SD)		*n*	% men	Mean age (SD)	
			Men	Women			Men	Women			Men	Women			Men	Women
2007	12,255	64.0	47.0 (10.4)	52.4 (12.8)	0	–	–	–	0	–	–	–	12,255	64.0	47.0 (10.4)	52.4 (12.8)
2008	11,317	65.3	47.1 (10.0)	52.1 (12.4)	0	–	–	–	0	–	–	–	11,317	65.3	47.1 (10.0)	52.1 (12.4)
2009	11,332	66.2	47.6 (9.9)	52.8 (12.1)	0	–	–	–	0	–	–	–	11,332	66.2	47.6 (9.9)	52.8 (12.1)
2010	9935	66.7	47.8 (10.2)	52.6 (12.0)	0	–	–	–	0	–	–	–	9935	66.7	47.8 (10.2)	52.6 (12.0)
2011	9565	67.1	48.2 (10.4)	54.1 (12.0)	1265	68.1	52.8 (9.0)	56.5 (10.3)	0	–	–	–	10,325	67.2	48.6 (10.4)	54.3 (11.9)
2012	7821	68.8	48.7 (10.7)	54.3 (12.5)	6037	65.4	51.9 (9.3)	57.3 (11.0)	0	–	–	–	12,488	67.4	49.9 (10.4)	55.6 (12.0)
2013	5640	71.8	47.7 (11.0)	54.5 (12.3)	3199	67.7	51.5 (9.6)	56.0 (11.6)	40	c	c	c	8382	70.5	48.9 (10.7)	55.2 (12.0)
2014	2969	74.8	47.9 (11.3)	55.0 (12.3)	474	70.9	50.1 (9.7)	53.4 (12.3)	8702	65.9	55.3 (9.2)	62.3 (10.9)	11,630	68.0	53.1 (10.5)	60.7 (11.7)
2015	536	83.4	38.7 (11.5)	49.1 (13.4)	35	c	c	c	14,650	64.4	54.9 (10.0)	62.1 (11.9)	15,189	65.1	54.2 (10.7)	61.9 (12.0)
Total^a^	53,794	66.4	47.1 (10.5)	52.7 (12.5)	10,260	66.5	51.8 (9.4)	56.7 (11.2)	22,565	64.8	55.1 (9.8)	62.2 (11.6)	72,277	65.6	48.9 (11.0)	55.0 (13.0)

*PEG-IFN* Pegylated Interferon α, *RBV* Ribavirin, *SD* standard deviation

^a^The total for the 2007–2015 period is less than the sum of the annual numbers of patients because some patients may have initiated successive HCV treatments during the period. The total number of patients initiating a treatment (*n* = 72,277) during the 2007–2015 period is less than the sum of numbers of patients by type of therapy because some patients may have initiated successive HCV treatments with different types of therapies during the period

^b^The annual number of patients initiating a treatment was less than the sum of the annual numbers of patients by type of therapy because some patients may have initiated successive HCV treatments with different types of therapies during the same year

^c^Data not available because of small numbers

Irrespective of the year and type of therapy, women were older than men at initiation, with a mean age of 55.0 vs. 48.9 years throughout the studied period. The increase in mean age observed in both genders between 2007 and 2015 was particularly marked in 2011–2012 and in 2014–2015, due to patients initiating either 1st-wave PI-based regimens or 2nd-wave DAAs, respectively.

**Fig. 4:**
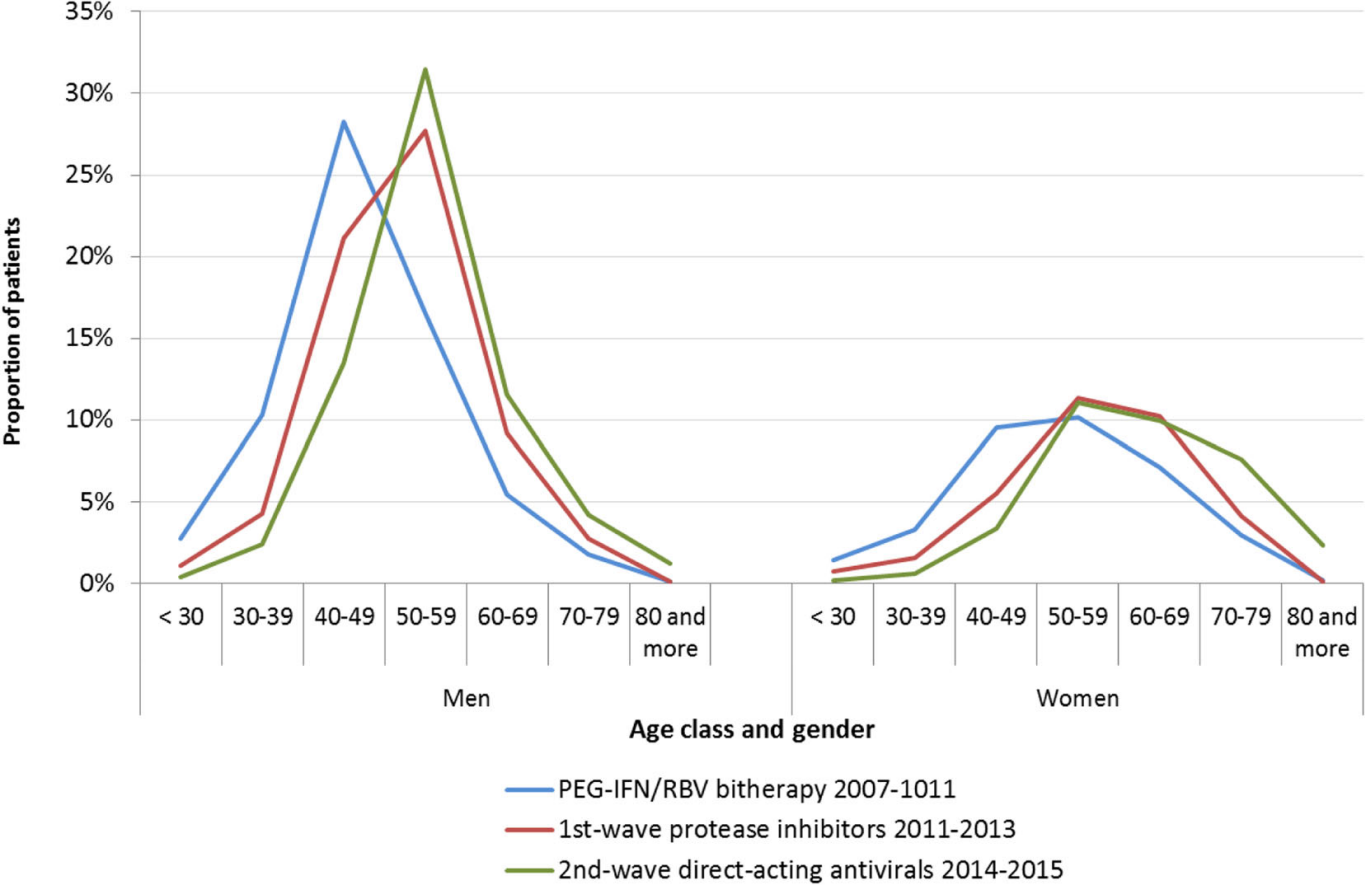
Distribution by age class and gender of patients initiating HCV treatment according to the type of therapy and the period, France, 2007–2015

Figure 4 illustrates the aging of patients with the type of therapy. For men, the age groups the more represented were 40–49 years for PEG-IFN/RBV bitherapy and 50–59 years for 1st-wave PIs and 2nd-wave DAAs. For women, 40–49 and 50–59 years were the age groups most represented for PEG-IFN/RBV bitherapy and 50–59 and 60–69 for 1st-wave PIs and 2nd-wave DAAs. The proportion of patients aged 60 years and over increased from 11% for bitherapy to 18% for 1st-wave PIs and 26% for 2nd-wave DAAs for men, and from 29% for bitherapy to 42% for 1st-wave PIs and 57% for 2nd-wave DAAs for women.

#### Second-wave DAAs in 2014–2015

Between 2014 and 2015, the proportion of men decreased and the distribution of age groups changed for both genders (Table 2) with an increase in the proportions of patients under the age of 40 (2.9% to 5.1% for men, 1.6% to 2.8% for women) and over the age of 79 (1.5% to 2.1% for men, 5.4 to 7.5 from women). We also observed a marked increase in the proportion of patients benefiting from CMUC (from 14.0 to 15.6%) and AME (from 1.1 to 2.0%), while the proportion of those benefiting from LTD status for HCV declined from 54.6 to 45.3%.

**Table 2:** Characteristics of patients initiating HCV treatment with 2nd-wave DAA-based regimens in 2014–2015, France

	2014				2015			
	Men	Women	Total		Men	Women	Total	
	%	%	n	%	%	%	n	%
Total	65.9	34.1	8702	100	64.4	35.6	14,650	100
Age group (years)^a^								
< 30	0.3	0.3	30	0.3	0.8	0.7	112	0.8
30–39	2.6	1.3	184	2.1	4.3	2.1	519	3.5
40–49	20.6	9.4	1461	16.8	20.5	9.6	2441	16.7
50–59	50.1	30.5	3776	43.4	48.2	31.8	6204	42.4
60–69	18.6	31.0	1985	22.8	17.6	27.1	3068	20.9
70–79	6.3	22.1	1015	11.7	6.5	21.2	1716	11.7
80 and over	1.5	5.4	249	2.9	2.1	7.5	585	4.0
Complementary Universal Health insurance (CMUC)^a^								
Yes	15.5	11.3	1218	14.0	17.3	12.6	2291	15.6
State Medical Assistance (AME)^a^								
Yes	0.9	1.4	93	1.1	2.1	1.9	298	2.0
Long-term disease status (LTD) for HCV^b^								
Yes	53.4	56.9	4276	54.6	43.2	48.9	6017	45.3
Previous HCV treatment initiation between 2007 and 2013								
No	52.5	59.8	4786	55.0	72.1	74.9	10,703	73.1
Yes^c^	47.5	40.2	3916	45.0	27.9	25.1	3947	26.9
*with PEG-IFN/RBV bitherapy only*	*72.5*	*72.1*	*2833*	*72.4*	*81.3*	*80.6*	*3200*	*81.1*
*with 1st-wave PIs only*	*12.6*	*13.6*	*506*	*12.9*	*9.3*	*10.9*	*388*	*9.8*
*with PEG-IFN/RBV bitherapy and 1st-wave PIs*	*14.9*	*14.3*	*575*	*14.7*	*9.4*	*8.5*	*359*	*9.1*

*PEG-IFN* Pegylated Interferon α, *RBV* Ribavirin, *1st- wave PIs* first-wave protease inhibitors

^a^Missing values: in 2014, 2 patients for age, 13 patients for CMUC and AME; in 2015, 5 patients for age

^b^This data was available only for beneficiaries of the general insurance scheme

^c^Previous HCV treatment initiation with 2nd- wave DAAs in 2013 for 2 patients

Among patients initiating DAA-based regimens in 2014, 45.0% had previously started a HCV therapy between 2007 and 2013 (men more often than women), among whom 27.6% with 1st-wave PI-based therapy. The proportion of patients previously treated sharply decreased in 2015 (26.9%), as well as the proportion of those with a history of 1st-wave PI treatment (18.9%).

#### PEG-IFN/RBV bitherapy in 2015

Despite treatment advances, 536 patients still initiated PEG-IFN/RBV bitherapy in 2015. Most were male (83.4%). As regards age, 57.5% of men and 25.8% of women were under 40 years old. This contrasts with those initiating a 2nd-wave DAA-based regimen (5.1% and 2.8%, respectively) in 2015 and those initiating PEG-IFN/RBV bitherapy in in 2010–2013 (13.2% and 3.8%, respectively). These 536 patients were twice as likely to benefit from CMUC as those initiating 2nd-wave DAAs (38.1% vs. 15.6%), and 17 times more likely to benefit from AME (35.8% vs. 2.0%). Conversely, 24.2% benefited from LTD status for HCV compared with 45.3% for patients treated with 2nd-wave DAAs in 2015. Nineteen percent of the 536 patients initiated a HCV treatment between 2007 and 2013.

## Discussion

For the first time in France, this work analyses trends in the implementation of successive HCV treatment advances and describes patients’ demographical characteristics over the last decade. From 2007 to 2015, we observe that approximately 10,000–15,000 patients initiated at least one HCV treatment each year (except in 2013) and that these numbers decreased each time the arrival of a new drug was expected. Our results highlight that 1st-wave PI and 2nd-wave DAA regimens have been rapidly implemented in France, in particular thanks to compassionate use programmes. They also demonstrate the large-scale diffusion of 2nd-wave DAA-based regimens, with 22,600 patients having initiated these therapies over the 2014–2015 period. In addition, both the patients’ characteristics and DAA combinations (including DAA combinations with PEG-IFN and RBV) suggest that the use of 2nd-wave DAA-based regimens in real life was in line with the French treatment guidelines [16, 32].

Trends in the annual numbers of patients initiating HCV treatment over the studied period may be the result of a combination of the following factors: the availability of the successive new therapies, the evolution of the knowledge on DAAs efficacy and tolerance, the epidemiological, clinical and virological characteristics of HCV-infected population, therapeutic guidelines and access to therapies. According to our data, approximately 10,000 patients initiated a regimen containing a 1st-wave PIs, mainly in 2012. The relatively poor uptake of 1st-wave PI regimens can be explained by the fact that they were recommended only for patients infected with GT1 and were restricted to patients with F4 liver fibrosis before MA. They were also probably used mainly for advanced disease after MA due to serious adverse events [33]. The imminent arrival of 2nd-wave DAAs may also have played a major role in the lower number of patients initiating 1st-wave PIs in 2013. The marketing authorisation for several 2nd-wave DAAs in 2014 led to increased numbers of patients initiating HCV treatment, with 8700 patients receiving 2nd-wave DAAs in 2014. This number was however lower than expected [16, 17], especially when compared with the annual numbers of patients who initiated PEG-IFN/RBV bitherapy in the late 2000s. High prices and restricted access to these drugs in France are probably the main cause for this, even though this lower than expected influx of patients in 2014 was also observed in Germany where access to DAAs has been universal from the start [34]. In line with the results for Germany, we observed that the number of patients initiating 2nd-wave DAAs reached a peak in the 1st quarter of 2015 and then decreased before finally stabilizing. This may be explained by the fact that a) the large majority of patients with advanced liver fibrosis eligible for DAA treatment [15] had already been treated and b) price reductions were anticipated. Other explanations for the lowered than expected number of patients initiating 2nd-wave DAAs are feasible. The annual numbers of patients who initiated PEG-IFN/RBV bitherapy in the late 2000s may be overestimated due to the definition we used for therapy initiation (no reimbursement for the same drug combination in the six previous weeks) and to frequent discontinuation of bitherapy because of adverse events. Conversely, the number of patients initiating DAAs in 2014 is possibly underestimated because of suboptimal coding of these drugs at the beginning of that year. An analysis performed by the main health insurance scheme in France, identifying drugs based on codes and costs, showed higher numbers of patients initiating 2nd-wave DAA based regimens for the two first quarters of 2014 [35]. The decrease in the prevalence of HCV chronic infection, from 232,000 in 2004 [36] to 193,000 in 2011 [18], and therefore in the pool of patients to treat, is certainly another explanation for the unexpected lower numbers of patients initiating DAAs in 2014.

Finally, almost 22,600 patients initiated a 2nd-wave DAA-based regimen between the last quarter of 2013 and the end of 2015, corresponding to approximately 20,300 patients whose infection was cured, assuming a SVR of 90%. This percentage takes into account the minimum SVR observed in real life in France [30, 31] and in other countries [37, 38], as well as the fact that data available did not indicate the recommended treatment duration for each patient. Accordingly, we could not verify whether patients completely adhered to their treatments. This estimated number of patients cured with DAAs in 2014–2015 corresponds to approximately 17% of persons with diagnosed chronic infection in France [18, 19]. This is very close to German estimates (24,000 patients cured among 160,000 patients with diagnosed viremic infection- i.e., 15%) [34] where, over the studied period, access to DAAs was universal and prices were quite close to those in France [10]. This fact would suggest therefore, that despite everything, restricted access to DAAs in France may have had a limited impact on access to 2nd-wave DAAs during the first two years after their arrival. Indeed, irrespective of different European polices on treatment access, the main limitation to accessing DAAs in 2014–2015 was their high cost. This is despite the fact that France specifically implemented a financial mechanism, based on a progressive contribution from the pharmaceutical companies commercializing these drugs, to limit HCV treatment-related expenses [39].

This individual data analysis enabled us to study patients’ characteristics. With almost two thirds of men and a higher mean age for women, the profile of patients initiating at least one treatment over the 2007–2015 period was consistent with French epidemiological data [40]. In addition, approximately 15% of those who initiated 2nd-wave DAA-based regimens in 2014–2015 (19% among patients under 60 years, data not shown) were beneficiaries of complementary universal health insurance, as opposed to 9.6% among the general population aged under 60 years in 2012 [41]. Low socio-economic status has been previously shown to be an important and independent predictor of HCV infection in France [36].

As expected, patient age increased with the initiation of more advanced therapies. Indeed, 1st-wave PIs and 2nd-wave DAAs were mainly used for patients with F4 liver fibrosis and patients with at least “severe F2” [15], respectively, and hence predominantly for the oldest patients. A higher proportion of individuals aged 70 or more was observed for those initiating 2nd-wave DAA-based regimens than for those initiating 1st-wave PIs. This is certainly thanks to their shorter durations, better efficacy and fewer side effects.

Among patients initiating 2nd-wave DAA-based regimens, the increase, between 2014 and 2015, in the proportions of patients aged under 40 years and over 79 years, as well as the decrease in the proportion of patients who had initiated HCV treatment between 2007 and 2013, suggests greater access to those with less advanced liver fibrosis and to elderly patients in 2015 than in 2014.

In 2015, despite treatment advances, approximately 500 patients still initiated PEG-IFN/RBV bitherapy. Most were under 40 years, which indicates that some may have had a liver fibrosis level lower than “severe F2”. In addition, these patients were characterized by high proportions of men and of beneficiaries of CMUC and AME, suggesting that a non-negligible proportion of them were migrants or low-income individuals, perhaps drug users and/or prison inmates. However, it was not possible to determine whether these individuals did not access DAAs because they did not meet the minimum stage of liver fibrosis required in 2015 (F2 “severe”) or because some of them may come from vulnerable populations. In any case, the number of patients initiating PEG-IFN/RBV bitherapy is expected to decrease in data from mid-2016 onwards thanks to universal access to DAAs announced by the French Health Ministry in 2016 [13]. Zimmermann et al. also found a non-negligible number of patients starting PEG-IFN/RBV in 2015, although they indicated that their methodology, based on aggregated data, did not allow them to specifically identify PEG-IFN/RBV bitherapy [34].

The main strength of our work is that it consisted in a complex analysis of individual data (all healthcare reimbursements for each beneficiary) from the national health insurance system databases [26], which cover almost the entire French population. Despite the availability of special insurance benefits which provide free access to healthcare (including DAAs) for vulnerable populations (i.e. beneficiaries of CMUC or AME), we cannot exclude the possibility that some marginalized individuals may not have been covered by the health insurance system because they did not complete necessary administrative formalities. However, these people were probably not already in care for hepatitis C and they surely could not have initiated costly HCV treatment. Health insurance scheme database information allowed us to identify each drug reimbursed for each beneficiary and to study the latter’s care path and certain socio-demographical characteristics. These databases are particularly well adapted to investigating HCV treatment because of the specificity of HCV drugs (or the association of PEG-IFN and RBV).

However, as the purpose of these databases is healthcare reimbursement, data on patient characteristics are limited and do not include detailed clinical data or results of para-clinical examinations, such as genotype, severity of liver fibrosis or HCV RNA. Another limitation is that the identification of the patients initiating a HCV treatment was based on drug code detection, and so was dependent on the quality of coding, which we suspect to be suboptimal for 2nd-wave DAAs in the first quarters of 2014. This explains why, for some patients, only DCV+/RBV (without SOF) was detected, mainly in the second quarter of 2014. In addition, the numbers of patients initiating DAA-based regimens may have been underestimated because they did not take into account patients enrolled in clinical studies or those whose treatment was donated by pharmaceutical companies. However, the impact on patients’ characteristics may be limited. Conversely, the annual numbers of patients who initiated PEG-IFN/RBV bitherapy in the late 2000s may be overestimated due to a) the 6-week delay between two deliveries for the same drug combination we used to define therapy initiation and b) frequent and long-time discontinuation of bitherapy because of adverse events. Our assumption about this maximum delay, based both on the data and on experience of hepatologists, seems to us the most appropriate to take into account possible variations, in the real-life, of the recommended delay (4 weeks) between two prescriptions and the context of highly supervised prescriptions of DAAs.

## Conclusions

France has seen the large-scale and rapid implementation of successive advances in HCV treatment. The numbers of patients who initiated 2nd-wave DAA-based regimens sharply increased between 2014 and 2015. With the announcement of universal access to DAAs in mid-2016 [13, 14] and price reductions, access to 2nd-wave DAAs is expected to expand even more. Universal therapy has recently been found to be the most effective strategy for reducing the 5-year incidence of cirrhosis, liver complications and liver deaths [42]. Universal access is also essential to reduce, through treatment-as-prevention, new HCV infections that mainly occur in people who inject drugs [43, 44]. Thus, unlimited access to DAAs constitutes a necessary (but not sufficient) condition to reach World Health Organization’s elimination targets, which include reducing HCV-related deaths by 65% and new HCV chronic infections by 90% by 2030 [45]. These goals cannot be achieved without a significant improvement in HCV screening effectiveness [46]. Complementing existing HCV risk-based testing strategies with additional population-based screening has been a topical issue in France in recent years [16, 46, 47]. A recent cost-effectiveness study based on French data has demonstrated that universal screening of all individuals aged 18 to 80 years could be the most effective and a cost-effective strategy, provided that antiviral treatment is rapidly initiated after diagnosis (S. Deuffic-Burban, submitted article). These results will be useful for the reassessment of screening strategy. Effective HCV screening rapidly followed by DAA treatment for all is the new challenge in France.

## Abbreviations

AME: State Medical Assistance
BOC: Boceprevir
CI: Confidence Interval
CMUC: Complementary Universal Health Insurance
CrI: Credibility Interval
CUP: Compassionate use programme
DAA(s): Direct-acting antiviral(s)
DCV: Daclatasvir
DSV: Dasabuvir
DU: Drug users
GT: Genotype
HCC: Hepatocellular carcinoma
HCV: Hepatitis C virus
ICD: International Classification of Disease
LDV: Ledipasvir
LTD: Long-term disease
MA: Marketing authorisation
NIR: National identification number
OBV: Ombitasvir
PEG-IFN: Pegylated interferon α
PI(s): Protease inhibitor(s)
PMSI: Programme for the medicalization of information systems
PTVr: Ritonavir-boosted Paritaprevir
RBV: Ribavirin
SMV: Simeprevir
SNIIRAM: French national health insurance system
SOF: Sofosbuvir
SVR: Sustained virological response
TVR: Telaprevir
